# How should cardiac xenotransplantation be initiated in Japan?

**DOI:** 10.1007/s00595-024-02861-7

**Published:** 2024-05-11

**Authors:** Shunsuke Saito, Shuji Miyagawa, Takuji Kawamura, Daisuke Yoshioka, Masashi Kawamura, Ai Kawamura, Yusuke Misumi, Takura Taguchi, Takashi Yamauchi, Shigeru Miyagawa

**Affiliations:** 1 Department of Cardiovascular Surgery, Osaka University Graduate School of Medicine, 2-2 Yamada-oka, Suita, Osaka 565-0871 Japan; 2 Department of Pediatric Surgery, Osaka University Graduate School of Medicine, Suita, Osaka 565-0871 Japan

**Keywords:** Cardiac xenotransplantation, Genetic engineering, Pig, Hyperacute rejection, End-stage heart failure

## Abstract

The world's first clinical cardiac xenotransplantation, using a genetically engineered pig heart with 10 gene modifications, prolonged the life of a 57-year-old man with no other life-saving options, by 60 days. It is foreseeable that xenotransplantation will be introduced in clinical practice in the United States. However, little clinical or regulatory progress has been made in the field of xenotransplantation in Japan in recent years. Japan seems to be heading toward a "device lag", and the over-importation of medical devices and technology in the medical field is becoming problematic. In this review, we discuss the concept of pig-heart xenotransplantation, including the pathobiological aspects related to immune rejection, coagulation dysregulation, and detrimental heart overgrowth, as well as genetic modification strategies in pigs to prevent or minimize these problems. Moreover, we summarize the necessity for and current status of xenotransplantation worldwide, and future prospects in Japan, with the aim of initiating xenotransplantation in Japan using genetically modified pigs without a global delay. It is imperative that this study prompts the initiation of preclinical xenotransplantation research using non-human primates and leads to clinical studies.

## Introduction

The world's first clinical cardiac xenotransplantation was performed on January 7, 2022, at the University of Maryland, using a genetically engineered pig heart with 10 gene modifications. A 57-year-old man with no other life-saving options survived for 60 days with this treatment [1]. Furthermore, experimental kidney and heart transplantation from genetically engineered pigs into human brain-dead cadavers at New York University showed that hyperacute rejection can be avoided [2]. Preclinical studies on organ transplantation using genetically modified pigs conducted in the U.S. in recent years have shown favorable results [3, 4]. Xenotransplantation will be introduced into clinical practice in the United States. However, although there has been steady development in basic research in the field of xenotransplantation in Japan, some of which is ahead of the world [5–9], there has been little clinical or regulatory progress. Japan seems to be moving toward a "device lag" [10] and the problem of over-importation of medical devices and technology [11] in the medical field still exists.

In this review, we summarize the necessity for and current status of xenotransplantation in the world, and the future prospects for xenotransplantation in Japan, which should be initiated, using genetically modified pigs, at the same time as the rest of the world.

## Need for cardiac xenotransplantation in Japan

In recent years, remarkable progress has been made in the treatment of severe heart failure, including drug therapy with the "fantastic four" [12] and left ventricular assist device (LVAD) therapy using HeartMate 3 (Abbot, Abbott Park, IL, USA) [13]. However, heart transplantation remains the definitive treatment for end-stage heart disease [14] and a shortage of heart transplant donors in Japan is a major obstacle. Although the number of heart transplantations performed in Japan is increasing each year, only 79 were performed in 2022 [15], whereas the number of patients on the waiting list for heart transplant registered with the Japan Organ Transplantation Network was 867 in November, 2023 [16]. The average waiting time in Status 1 for the 79 heart transplant recipients in 2022 was 1769 days [15].

In Japan, where the waiting period for heart transplantation is long, LVADs are particularly important as a bridge to transplantation. Recently, the use of LVADs as the main treatment for patients who are not candidates for heart transplantation has become increasingly important. This LVAD treatment is called ‘a destination therapy’. In the United States, 78.1% of patients with LVAD implantations do not subsequently undergo heart transplantation [13]. The short-term outcomes of the latest magnetic levitation LVAD (HeartMate 3) are good, with reported 1- and 2-year patient survival rates of 85.9% and 78.8%, respectively [13]. According to data from the Japanese registry for Mechanically Assisted Circulatory Support, the 1- and 2-year patient survival rates for implantable LVADs in Japan are 93% and 90%, respectively [17]. However, the 10-year long-term outcomes of LVAD implantation remain unknown, and the complications associated with LVAD treatment cannot be ignored. Complications include bleeding and infection, which occur in 35% and 55% of patients, respectively [18]. Right heart failure is another important complication, occurring in approximately 30% of patients [19]. Right heart failure is a poor prognostic factor for LVAD, and complications of severe right heart failure decrease the chances of heart transplantation [20]. No implantable devices for right heart assistance are covered by health insurance, and patients with a biventricular assist device (BiVAD) using extracorporeal right heart support cannot be discharged from hospital. Although implantable BiVADs using two implantable devices for left heart assistance have been reported [21, 22], a retrospective study on 93 implantable BiVADs identified patient survival rates of 56% and 47% at 1- and 2 years, respectively, which were worse than those achieved by LVADs [23]. In another recent study, the median 1-year survival rate was 58.5%, with a median pump thrombosis rate of 31% (mainly right-sided) [24]. The outcomes of totally artificial hearts were worse than those of BiVADs [25].

Recently, cardiac tissue engineering has been used in drug discovery and human disease modeling, with the subsequent clinical applications of cardiac regeneration therapy using induced pluripotent stem cells [26, 27]. Moreover, 3D bioprinting has been used successfully to create cardiovascular structures, substituting blood vessels, heart valves, and myocardium [28]. However, the creation of a perfect substitute heart has not been achieved, and its realization is likely to take decades.

**Fig. 1:**
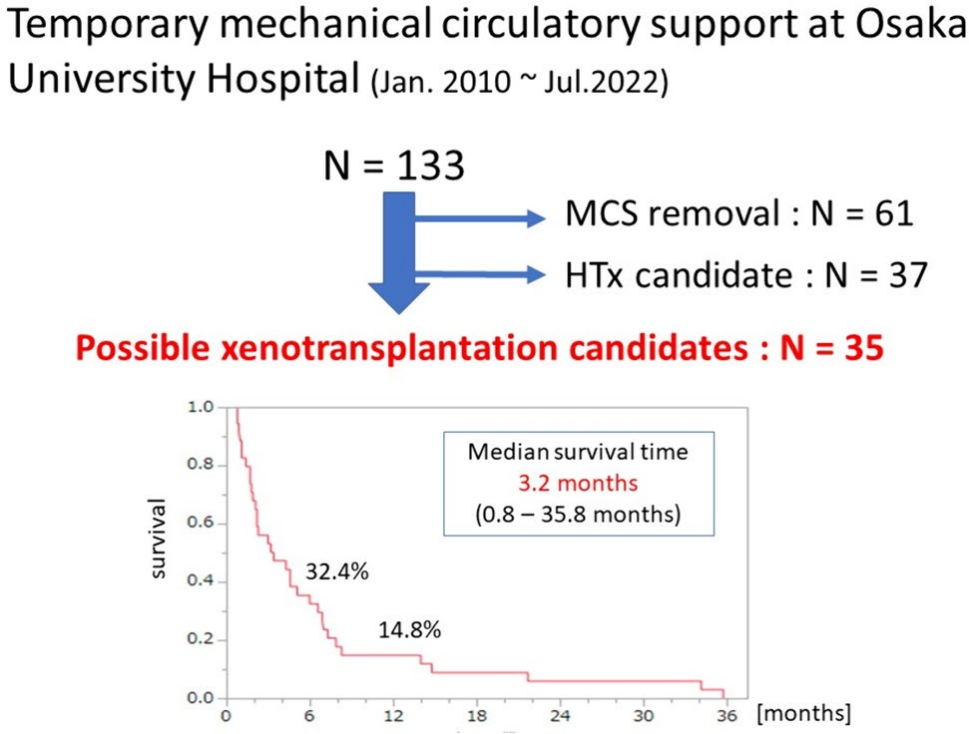
Between January, 2010 and July, 2022, a total of 133 patients required temporary mechanical circulatory support at Osaka University Hospital. Of these 133 patients, 35 were not candidates for human allotransplantation or left ventricular assist devices as destination therapy and could not be weaned off mechanical support. The clinical outcomes of these 35 patients were poor; however, such patients are possible candidates for cardiac xenotransplantation. *HTx* heart transplantation with human hearts, *MCS* mechanical circulatory support

For these reasons, cardiac xenotransplantation, defined as the use of animal hearts for transplantation, is expected to become a viable alternative to the allotransplantation of hearts from humans. Between January, 2010 and July, 2022, 133 patients required temporary mechanical circulatory support such as venoarterial extracorporeal membrane oxygenation (V-A ECMO), temporary extracorporeal ventricular assist devices, and Impella (Abiomed, Danvers, MA, USA) at Osaka University Hospital (Fig. 1). The temporary device was removed following functional recovery in 61 of these patients, and 37 patients were approved as heart transplantation or destination therapy-LVAD (DT-LVAD) candidates by the institutional committee. The remaining 35 patients were not candidates for human heart allotransplantation or DT-LVAD and could not be weaned off mechanical support. The clinical outcomes of these patients were poor, with a median survival of only 3.2 months (0.8–35.8 months). Such patients are potential candidates for cardiac xenotransplantation.

## History of clinical cardiac xenotransplantation

The world's first clinical xenotransplantation was conducted before Barnard performed the first human heart allotransplantation in 1967 [29]. In 1964, Hardy transplanted a chimpanzee heart into a human (Table 1). However, the transplanted heart was unable to assist the recipient's circulation sufficiently [30]. Subsequent attempts at transplantation using non-human primates (NHPs), sheep and pig hearts also failed [31–34]. Then, in 1984, Baily transplanted a baboon heart into a female neonate (Baby Fae), who survived for 20 days [35]. This greatly stimulated the subsequent development of pediatric cardiac allotransplantation. Subsequently, Religa [36] and Baruah [37] performed heart transplants from pigs in 1992 and 1996, respectively, and documented survival of 23 h and 7 days, respectively. Clinical cardiac xenotransplantation was not attempted thereafter for a long time [31].

**Table 1:** History of clinical cardiac xenotransplantation

Year	Surgeon or team (location)	Organ donor	Survival (day)	References
1964	Hardy (USA)	Chimpanzee	< 1	[30]
1968	**Ross (UK)**	**Pig**	** < 1**	[31]
1968	Cooly (USA)	Sheep	< 1	[32]
1969	Marion (France)	Chimpanzee	< 1	[33]
1977	Barnard (South Africa)	Chimpanzee	4	[34]
1984	Bailey (USA)	Baboon	20	[35]
1992	**Religa (Poland)**	**Pig**	** < 1**	[36]
1996	**Baruah (India)**	**Pig**	**7**	[37]
2022	**Griffith/ Mohiuddin (USA)**	**Pig (genetically engineered)**	**60**	[1, 90]
2023	**Griffith/ Mohiuddin (USA)**	**Pig (genetically engineered)**	**40**	[38]

Bold indicates the use of pigs as organ donors

On January 7, 2022, cardiac xenotransplantation of the heart of a genetically engineered pig was performed at the University of Maryland [1]. This case is discussed in detail in the following section. The second procedure was performed on September 20, 2023 [38].

## Usefulness of pigs as xenotransplant donors

NHPs, which are more closely related to humans immunologically, are advantageous as xenograft donors. In previous clinical xenotransplants, only those using baboon hearts avoided hyperacute rejection [35]. However, beyond the fact that the organs of NHPs are difficult to obtain, their use in human transplantation is not ethically acceptable.

Although pigs are immunologically discordant to humans, they are considered useful and viable xenotransplant donors for the following reasons [39]:

- Available in size and function equivalent to a human heart.
- High reproductive performance (high fecundity), short developmental time to sexual maturity and adult size allow for efficient multiplication.
- Ethical acceptance for the use of organs to save human lives.
- The natural lifespan of a pig is approximately 15–20 years, and a comparable lifespan of an organ can be expected.
- Pigs can be bred under designated pathogen-free (DPF) conditions to reduce the risk of disease transmission.
- Efficient and accurate gene modification techniques have been established to overcome immunological problems.

## Immunological issues in transplanting pig organs into humans

All humans and NHPs possess natural antibodies against the surface antigens of pig cells. When porcine organs are transplanted into humans or NHPs, these natural antibodies immediately bind to the vascular endothelial cells of the graft. Some activate the complement pathway (antibody-mediated complement activation), whereas others attract leukocytes that adhere to and infiltrate through Fc-receptor-mediated and Fc-independent mechanisms. This reaction usually occurs within minutes to hours [40] and is called hyperacute rejection (HAR). The histopathological features of hyperacute rejection include venous thrombosis, loss of vascular integrity, interstitial hemorrhage, edema, and innate immune cell infiltration [41–43].

The natural antigens responsible for HAR have been found to be against carbohydrate antigens on the surface of porcine cells. Mammals other than humans, apes, and old world monkeys have a glycosyltransferase called alpha1,3-galactosyltransferase (*alpha1,3GT* or *GGTA1*) [44], and this enzyme causes the expression of galactose-α(1.3)-galactose (αGal) on the cell surface. Humans, apes, and old-world monkeys have lost the *GGTA1* gene; therefore, they do not express αGal on the cell surface. Primates are believed to acquire natural antibodies against αGal when microorganisms colonize their gastrointestinal tracts during infancy because these microorganisms have the same surface antigens on their cells [45]. More recently compared with the loss of the *GGTA1* gene (approximately 3.5 million years ago), humans have lost the function of another enzyme involved in sialic acid synthesis: cytidine monophosphate-*N*-acetyl-neuramic acid hydroxylase (*CMAH*) [46], resulting in the absence of N-glycolylneuraminic acid (*Neu5Gc*). However, *Neu5Gc* is expressed on glycoproteins and glycolipids in most organs and cells of mammals, including pigs, and the diet-based intake of *Neu5Gc* provokes natural immunization and the production of anti-Neu5Gc antibodies in human serum. [47, 48]. Moreover, NHPs have natural antibodies against the sugar chain corresponding to the human Sd(a) blood group antigen synthesized by the β-1,4-*N*-acetyl-galactosaminyl transferase 2 (*β4GalNT2*) gene [49, 50]. Conversely, most humans have the Sd(a) antigen, and it is unclear whether the Sd(a) antigen produced by pig *β4GalNT2* shows antigenicity in most humans [51].

Although the most important mechanism in hyperacute rejection is antibody-mediated complement activation, it is known that the complement itself can recognize and kill xenogeneic cells thorough an alternative pathway [52]. Cells of the innate immune system such as natural killer (NK) cells, macrophages, and neutrophils also play a more important role in xenotransplantation than in allotransplantation [9].

## Modification of porcine cell surface antigenicity by gene modification technology

The first genetic alterations in pigs focused on human complement regulatory proteins (CRPs), such as membrane cofactor protein (MCP: CD46), decay-accelerating factor (DAF: CD55), and CD59 [8]. First, DAF transgenic pigs were produced in 1994, followed by transgenic pigs expressing other CRPs [53].

**Fig. 2:**
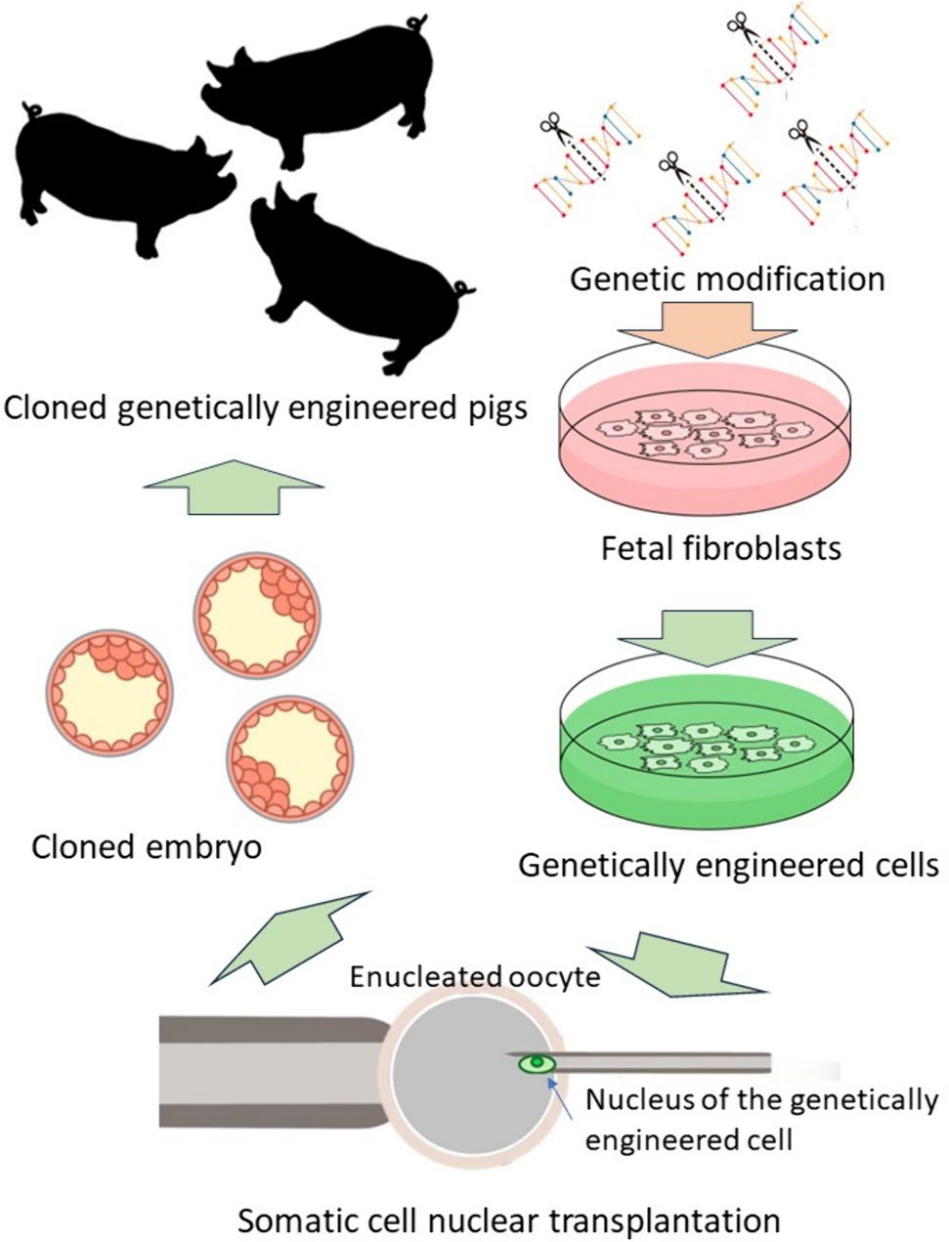
Somatic cell cloning is a technique in which the nuclei of cultured somatic cells are implanted into the cytoplasm of unfertilized eggs to create cloned embryos, and cloned individuals are born through a borrowed abdominal pregnancy. By applying procedures such as gene transfer or gene knockout to cultured somatic cells, genetically modified cell nuclei can be obtained. Using such nuclei to create cloned embryos leading to cloned individuals, a theoretically unlimited number of genetically modified individuals can be produced as required

On the contrary, even after *αGal* was found to be the main cause of HAR, the knockout of αGal was not easy. This is because unlike in mice, porcine embryonic stem cells have not yet been established. Therefore, competitive inhibition and remodeling of Gal epitopes by the overexpression of α1,2fucosyltransferase, α2,3sialyltransferase [54], β-D-mannoside β-1,4-*N*-acetylglucosaminyltransferase III (*GnT-III*) [5] and others were investigated. In 2002, the technology of "Dolly," a cloned sheep, was applied to develop an *αGal* knockout pig by combining gene engineering and nuclear transplantation technologies into fetal fibroblasts [55] (Fig. 2). This technology was used to eliminate the major carbohydrate antigens on porcine cells, αGal, Neu5Gc, and Sd(a) epitopes, which are the targets of human natural antibodies. Three genes, *GGTA1* [56], *CMAH* [57, 58], and *β4GalNT2* [59], were removed and so-called triple-knockout (TKO) pigs were created. Significantly lower binding of natural antibodies to TKO pig-derived cells than to wild-type pig cells in human blood has been confirmed [60].

Once the anti-pig antibody targets are eliminated, antibody-dependent cellular cytotoxicity (ADCC) by NK cells is attenuated. However, there is direct human NK cell cytotoxicity against pig cells because human NK cell receptors cannot bind to swine leukocyte antigen (SLA)-I. To address this issue, it is possible to suppress human NK cell activity by expressing human leukocyte antigen (HLA)-E on pig cell surfaces [61, 62]. Human macrophages are also activated by porcine cells. This is because CD47 on pig cells cannot bind to the “don't eat me” signal regulatory protein alpha (SIRPα) on human macrophages. Therefore, TG pigs expressing human CD47 were generated, and these pig cells were reported to be protected from human monocyte- or macrophage-mediated cellular cytotoxicity [63, 64]. Several other molecules that regulate monocytes/macrophages have been identified over the past decade. For example, HLA-G1 [65] and HLA-E [66] were found to inhibit monocytes/macrophages and NK cells. Monocytes and macrophages share many receptors in common with NK cells. Alterations in carbohydrate antigens, such as the overexpression of α2,6 sialic acid, are also effective in controlling monocyte/macrophage [67, 68]. Moreover, it has been reported that CD31 and CD177 can be used to control neutrophils [9].

## Methods to control acquired immunity

In pig-to-human heart transplantation, even if hyperacute rejection by natural antibodies, acute humoral xenograft rejection, and innate cellular xenograft rejection by innate immune cells can be prevented, adaptive cellular xenograft rejection, in which T- and B cells play major roles, is expected. The activation of human/NHP T cells in pig organs occurs either directly by antigen-presenting cells (APCs) of the pig or indirectly by APCs of human/NHPs. Several co-stimulatory and co-inhibitory signals are involved in this process [39]. The direct activation of T cells can be attenuated by the removal or downregulation of SLA molecules, which are the major histocompatibility complexes in pigs [69, 70]. Moreover, the blockade of co-stimulatory signals of T cell activation has been found to be effective. Among them, the CD40-CD154 co-stimulation blockade is considered important in pig-to-human/NHP transplantation. Since 2000, anti-CD154 monoclonal antibodies have been used in xenotransplantation experiments [71]; however, since anti-CD154 monoclonal antibodies are thrombogenic in humans, anti-CD40 monoclonal antibodies-based regimens have been established and shown good results for pig-to-baboon heterotopic [72] and orthotopic heart transplantation [3, 73].

## Measures against disorders of the coagulation system

Another facet of the pathobiology of pig organ xenotransplantation is dysregulation of the coagulation pathway [74, 75]. The contributing mechanisms include the human immune response to porcine organs, which triggers inflammation, vascular injury, and the procoagulant surface of pig endothelial cells. The molecular incompatibility of coagulation regulators among pigs, humans, and NHPs is also important. Thrombotic microangiopathy (TM) can occur and cause organ damage, despite immunological adjustments to avoid hyperacute xenograft rejection and systemic anticoagulation therapy [76, 77]. The complex of thrombomodulin (TBM) and thrombin encounters protein C flowing in the blood and converts this protein into activated protein C. Activated protein C breaks down activated factor VIII (VIIIa) and activated factor V (Va) produced in the coagulation reaction, and fibrin is no longer formed, thus halting fibrin clot formation. Pig TBM is unable to bind to human thrombin and activate human protein C; therefore, coagulation inhibition does not occur, whereas TM does. The expression of human TBM in donor pig cells can circumvent TM [78]. Furthermore, the function of human TBM is enhanced by the expression of the human endothelial protein C receptor [79].

## Other genetic modifications

In addition to the above genetic modifications of the immune and coagulation systems, the expression of anti-inflammatory proteins, such as human TNF-alpha-induced protein 3 (TNFAIP3, also known as A20) [80] and human heme oxygenase 1 (HMOX1) [81], have been found to be effective in protecting xenografts. The harmful overgrowth of pig hearts has been observed consistently in pig-NHP xenotransplantation in preclinical studies [73]. One solution to this problem is to create donor pigs with a loss-of-function mutation in the growth hormone receptor (*GHR*) gene [82, 83]. In orthotopic cardiac xenotransplantation between pigs and NHPs, *GHR* knockout improved outcomes, with 9 months of recipient-animal survival documented [3, 84].

## Xenozoonosis control

Genetically modified donor pigs can be maintained in DPF facilities to achieve a high microbiological and virological safety profile [85, 86]. Furthermore, highly sensitive and specific testing methods have been established for specific pathogens that should not be present in donor pigs [87]. In Japan, Otabi et al. developed a panel consisting of 76 highly sensitive polymerase chain reaction (PCR) detection assays that could screen 41 viruses, 1 protozoan, and a broad range of bacteria [88]. Among pig-specific pathogens, porcine cytomegalovirus (pCMV) has been reported to compromise graft survival [89]. In the University of Maryland case, pCMV activation was mentioned as a possible cause of transplanted organ failure [90]. Moreover, porcine endogenous retroviruses *(PERVs*) are integrated into the porcine genome. However, PERV infection has not been detected in numerous preclinical xenotransplantation studies in NHPs or other species, in in vivo infection experiments in different species [91], or in clinical xenotransplantation of porcine islet cells in patients with diabetes [92, 93]. Attempts to knock out all *PERVs* from the porcine genome using clustered regularly interspaced short palindromic repeats/CRISPR-associated proteins (CRISPR/Cas9) technology have already been successful [94, 95].

## Review of the case at the Maryland University

The patient was a 57-year-old man with mild chronic thrombocytopenia, hypertension, non-ischemic cardiomyopathy, and a history of mitral valve repair [1, 90]. He was hospitalized for severe heart failure with a left ventricular ejection fraction of 10% and required extensive intravenous inotropic support, which was escalated to intra-aortic balloon pumping and V-A ECMO. His case was reviewed by two regional and two prominent national heart transplantation programs, but the request for a transplant or LVAD was denied by all four programs because of poor adherence to treatment. The Food and Drug Administration approved cardiac xenotransplantation as an expanded access; that is, a potential pathway for a patient with a serious or immediately life-threatening disease or condition to gain access to an investigational medical product (drug, biological, or medical device) for treatment outside clinical trials.

Table 2 summarizes the 10 gene edits applied to the donor pigs. The source animal was derived from a PERV–C–negative line and tested every 3 months for pathogens that affect porcine or human health, including PERV-A, PERV-B, PERV-C, pCMV, and porcine lymphotropic herpesvirus. Table 3 lists the immunosuppressive drugs used in this patient.

**Table 2:** Ten gene modifications used in the Maryland University case

Genetic modification	Mechanism
Anti-immunogenic	
α-1,3-galactosyltransferase knockout (*GGTA1-KO*)	Deletion of Galactose-α-1,3-galactose (αGal)
Cytidine monophosphate-*N*-acetylneuraminic acid hydroxylase knockout (*CMAH-KO*)	Deletion of N-glycolylneuraminic acid (Neu5Gc)
β-1,4-*N*-acetyl-galactosaminyl transferase 2 knockout (*B4GALNT2/B4GALNT2L-KO*)	Deletion of blood group Sd(a) antigen
Reduce intrinsic graft growth	
Growth hormone receptor knockout (*GHT-KO*)	Reduction of downstream insulin growth factor-1 (IGF-1) signaling
Complement regulation	
Human membrane cofactor protein transgenic (*hCD46-tg*)	Suppression of human complement activity by mediating cleavage of C3b and C4b complement deposition
Human decay-accelerating factor transgenic (*hCD55-tg*)	Decay of C3 and C5 convertase formations and downstream complement activation
Prevent macrophage activation	
Human signal regulatory protein alpha transgenic (*hCD47-tg*)	Prevention of macrophage activation
Anti-coagulation	
Human thrombomodulin transgenic (*hTBM-tg*)	Binding of human thrombin and activation of Protein C via activated thrombin
Human endothelial protein C receptor transgenic (*hEPCR-tg*)	Activation of Protein C
Anti-inflammatory	
Human heme oxygenase 1 transgenic (*hHMOX1-tg*)	Decrease in oxidative products

**Table 3:** Immunosuppressive drugs used in the University of Maryland case

Immunosuppressive drug	Mechanism	Remarks
Induction		
Anti-thymocyte globulin (ATG)	Removal of T cells	
Anti-CD20 monoclonal antibody (Rituximab)	Removal of B cells	
Berinert	Complement suppression (human C1-inactivator)	
Maintenance		
Anti-CD40 monoclonal antibody (KPL-404)	IgG4 monoclonal antibody, binds to CD40, inhibits T cell-dependent B cell immune response	Currently not available in Japan
Mycophenolate mofetil (MMF)	Nucleic acid synthesis inhibitor	
Tacrolimus	Calcineurin inhibitor	Only used as an alternative when MMF could not be used because of neutropenia
Support		
Steroids	Anti-inflammatory	
Intravenous immunoglobulin (IVIG)	Regulation of immune response and immunoglobulin supplementation	Used on day 43 for hypogammaglobulinemia and on day 50 when patients condition deteriorated. These are suspected to have been a cause of graft dysfunction
Ecluximab (anti-C5 monoclonal antibody)	Complement suppression	Used only on day 54

During the transplantation procedure, the patient suffered aortic dissection from the aortic cross clamp and underwent repair. The residual dissection caused an occlusion of an upper-pole left renal artery and an endovascular stent was placed postoperatively. However, acute renal failure persisted, and renal replacement therapy was required. The chest was closed on day 2 after the transplantation and the patient was extubated. ECMO was discontinued on day 4, and the Swan-Ganz catheter was removed on day 6 with stable hemodynamics. On day 12, exploratory laparotomy was required but no signs of acute ischemia or perforation was observed. Because of the complicated postoperative course, the first endomyocardial biopsy was not performed until postoperative day 34, which revealed no evidence of rejection. The patient was rehabilitated without any cardiovascular support, and the xenograft functioned normally without evidence of rejection. On day 43, hypotension developed, and the patient was reintubated. Quantitative PCR was performed on peripheral blood mononuclear cells, which tested positive for pCMV. The patient had hypogammaglobulinemia; therefore, intravenous immunoglobulin was administered, and the antiviral therapy was changed from ganciclovir to cidofovir. The patient was extubated on day 47. On day 49, the patient’s hemodynamics suddenly deteriorated, and VA-ECMO was restarted. Echocardiography showed a dramatic increase in left and right ventricular wall thicknesses. Repeated endomyocardial biopsy was negative for pathologic antibody-mediated rejection on day 50, but positive on day 56. The xenograft dysfunction did not improve, and life support was withdrawn on day 60 after transplantation.

After thorough investigation, the transplantation team concluded that there were three possible causes of graft dysfunction: endogenous xenoantibody-mediated rejection, the exogenous administration of intravenous immunoglobulin -containing xenoantibodies, and reactivation of pCMV within the xenograft [90].

## Current situation and prospects in Japan

Japan has led the world in basic research on xenotransplantation. When the mechanism of rejection in xenotransplantation was still unknown, Miyagawa et al. conducted a study using a guinea pig-to-rat heterotopic heart transplantation and concluded that the alternative pathway of complement activation is involved in the hyperacute rejection that occurs in this discordant combination. He reported that this response was related to the species specificity between complement and CRPs, which triggered a surge of research in the field of xenotransplantation worldwide (8). Since then, world-leading results have been reported from Japan, especially in the fields of complement, innate immunity, and glycan research (5–9).

Conversely, there has been no remarkable progress in clinical studies and regulation. In Japan, the ‘Regenerative medicine promotion act’ was passed and promulgated in 2013 and introduced the same year. The “Act on the safety of regenerative medicine” and the “Revised pharmaceutical affairs act” were passed and promulgated in 2013, and introduced in 2014 [96]. The ‘Act on the safety of regenerative medicine’ covers three classes of risk-dependent procedures: high-risk (Class I), medium-risk (Class II), and low-risk (Class III). Class I covers procedures involving human embryonic stem cells, induced pluripotent stem (iPS) cells, cells like iPS cells, cells in which a gene is introduced, and xenogeneic and allogeneic cells. Thus, the “Act on the safety of regenerative medicine” and the “Revised pharmaceutical affairs act” cover xenogeneic cell transplantation, including islet xenotransplantation, but not xenogeneic organ transplantation. The regulation of xenogeneic organ transplantation is currently under discussion. In the near future, xenogeneic organs, such as the heart and kidneys are expected to be treated as “regenerative medical products.”

The Research and Development Division, Health Policy Bureau, the Ministry of Health, Labour and Welfare (MHLW), updated the “Public Health Guidelines on Infectious Disease Issues in Xenotransplantation” in 2016, from the original 2001 publication [96]. The objective of this study was to prevent infections and the spread of emerging infectious diseases caused by xenotransplantation, making it relevant to public health.

Clinical trials are ready to begin in some areas of the world, whereas Japan seems to be behind other countries. Regarding regulation, there has been no progress since the “Public Health Guidelines on Infectious Disease Issues in Xenotransplantation” was revised in 2016 and the “Act on the Safety of Regenerative Medicine” was introduced in 2014. However, substantial research funds from the Japan Agency for Medical Research and Development have been allocated for islet xenotransplantation [97]. In February, 2022, an informal meeting was held between the MHLW and the Japanese Society for xenotransplantation to provide information on clinical trials in the United States and discuss the need for laws and regulations in xenotransplantation in Japan. Subsequently, an MHLW study group was formed and discussions on the formulation of regulations specifically for xenotransplantation were initiated [98]. The Japanese Government is currently focusing on clinical xenotransplantation. We hope that the Japanese government will provide large grants for research on xenotransplantation of the heart, kidneys, and other organs soon. It is imperative that this study prompts the initiation of preclinical xenotransplantation research using NHPs and leads to clinical studies.
